# Customized Incubation Services and Growth of Tenants: The Mediating Effect of Behavior Orientation of Social Networking Services

**DOI:** 10.3389/fpsyg.2021.764168

**Published:** 2021-12-24

**Authors:** Li Zhang, Yongtao Zhou

**Affiliations:** Department of Business Administration, Business School, Hubei University, Wuhan, China

**Keywords:** business incubator, incubation services, customized incubation services, tenants’ growth, social networking services

## Abstract

The third-generation incubator is generally characterized by embedment of social networking and customized incubation services are closely embedded in the social networking. How the social networking play their role in the process that customized incubation services facilitate the growth of tenant? In order to disclose whether social networking services (SNS) mediate the impact of customized incubation services on the growth of tenants, this article focuses on the whole process where customized services facilitate the growth of tenants by means of social networking services. First, it employs situational theory and contingency theory to analysis why customized services result in behavior of social networking services; second, it explores why behavior of social networking services facilitates the growth of tenants based on co-production theory and social network theory; next, it conduct a study on the direct relationship between customized services and growth of tenants anchoring on the theory of co-production, customer satisfaction, and dynamic environment. Based on these theories, it develops the overall theoretical model of mediating effect. Following that, it conducts empirical test: it has first ascertained whether there is a positive relationship between customized services and growth of tenants. Then, three paths of the theoretical model have been measured by means of the structural model. At the same time, the *t*-test and the Sobel test are employed to justify their significance. If we only contemplate customized incubation services and growth of tenants, they are positively correlative. On the other hand, if referring to the role of social networking services in this process, we disclosed that not only customized incubation services positively impact behavior of social networking services, but also behavior of social networking services positively facilitates the growth of tenants; at the same time, the customized incubation services exhibit no direct impact on the growth of tenants otherwise. It witnesses that the behavior of social networking services fully mediates the relationship between customized services and the growth of tenants. As a result, we should promote incubation services to be deeply embedded in social networking services; incubator management should even improve the capability to deal with big data embedded in social networking services. In additional, entrepreneurial ecosystems should be also embedded in social networks intensively.

## Introduction

There is no generally accepted definition of “social networking services.” While according to Wikipedia, a social networking service is an online platform, which people use to build social networks or social relationship with other people who share similar personal or career interests, activities, backgrounds, or real-life connections. There is a variety of social networking services available online. However, most incorporate common features are: (1) Social networking services are Web 2.0, Internet-based applications, (2) User-generated content (UGC) is the lifeblood of social networking services, (3) Users create service-specific profiles for the site or app that are designed and maintained by the social networking services (SNS) organization, and (4) Social networking services facilitate the development of online social networks by connecting profile of a user with those of other individuals or groups^1^. Social networks are essentially information networks. People use the social networking services not only for viewing and obtaining information, but also for creating and maintaining social relationships. Social networking services have fundamentally changed how people communicate with each other. More importantly, they have made it extremely easy to establish relationships among individuals as well as between individuals and organizations. Social networking services are also referred to as social networking site or SNS or social media. These terms are used interchangeably in this article.

The first-generation business incubators mainly provide physical room to their tenants. The second-generation incubators mainly provide intangible valued services such as financial services, entrepreneurial mentoring, and technical assistance. The third-generation incubators focus on providing multiple accesses to external resources and knowledge (Pauwels et al., 2016). Owing to their lack of knowledge or competence, tenants are often confused to make a choice when facing multiple solutions. Thus, customized services that could meet the specified demands of tenants become a new trend of incubator operation. More and more incubators have developed the operation of customized services.

Customized services are accompanied by wide information dissemination, large-scale exploratory learning, and cross-fertilization of online and offline behaviors. Hence, an intuitionistic phenomenon is that customized services are deeply embedded in social networking services. For example, social networking is employed a primary tool to propagate potential as well as financial demand of tenant; at the same time, tenants search possible investors mainly by means of social networking. Entrepreneurial mentor could have been advanced promptly once their communication has been set in the context of social networking. Technology assistance might have been more convenient, if the technology transfer, especially explicit knowledge, has matched the social networking.

Comparing with the first-generation and second-generation incubator, the third-generation incubator would mainly offer the accesses to heterogeneous networks. It is called a kind of networked incubation. Those networks facilitate tenants to contact with service providers directly, so that to express their demands and gain specific services. As we know, the foundation of being able to express demands and gaining services is the convenient flow of information. In the case that SNS is on hand, whether the appeal to customized incubation services unconsciously drive the process of co-production conducted by SNS remains unclear. In face of the context of customized incubation services, behaviors of SNS tightly embedded in those services as well as the growth of tenant, we could not help speculating what role the SNS has played in the process that customized incubation services might facilitate the growth of tenant? If the customized incubation services have impacted the growth of tenant by means of social networking, why the customized incubation services result in the behavior of SNS? At the same time, why the behavior of SNS impact the growth of tenant? When researchers, management, as well as policymaker contemplate the customized incubation services extensively embedded in the SNS, they could not help speculating the theoretical logics underpinned those three behaviors. The motivation of this article is just here to make clear the theories, which could account for the role of SNS when customized incubation services are conducted to facilitate the growth of tenant.

Then, the study questions are as follows: did customized services result to behavior orientation of social network services? Did behavior orientation of social network services facilitate the growth of tenants? Extant study shows that customized services have a positive effect on the growth of tenants (Vanderstraeten et al., 2016), thus did behavior orientation of social network services play a mediating role between customized services and growth of tenants?

As the article highlights the role of social networking in the casual relationship between customized services and growth of tenant, its theoretical contributions are: (1) Developing theory that behaviors orientation of social networking services facilitates the growth of tenant and (2) Developing the theoretic model of mediating role of behaviors orientation of social networking services.

The rest of this article is organized as follows. Section “Theoretical Analysis and Hypotheses” describes theory analysis and hypotheses. Section “Methodology” describes data, measurement, and methodology. Section “Data Analysis and Hypothesis Testing” describes conclusion and management implications.

## Theoretical Analysis and Hypotheses

**FIGURE 1:**
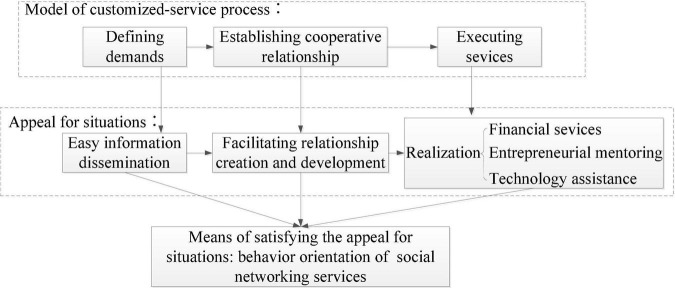
Trail of exploiting the relationship between customized service and behavior orientation of social networking services.

The relationship of customized services and behaviors orientation of social networking services is developed based on situational theory. According to the theory, learning and development only occur in communities of practice. This concept implies that people who engage in common endeavors must not simply learn, but create a community environment that facilitates creative learning (Wenger, 1998, p. 22). Thus, we disclose the microprocesses of customized service anchoring on situational theory and then it makes clear the relationship between customized service and behavior orientation of social networking services (Figure 1).

Customized incubation services differ from universal incubation services; they are processes of providing idiosyncratic services to satisfy specific needs. Such processes consist of three independent subprocesses: defining the needs of tenants, establishing cooperative relationships, and realizing incubation services.

According to situational theory (Theodorakopoulos et al., 2014), parties involved in co-production should create situations that facilitate information dissemination, spark development of cooperation, and push the process of financial services, entrepreneurial mentoring, and technical assistance, while social networking services are appropriate means to create the very situations.

First, social networking services create situations that facilitate information dissemination. Social networking services enable users to directly transmit information using functions embedded in social media software such as passive connecting with others, exhibiting contents, responding to others, and comment at any time. So, parties involved in co-operation could disseminate real-time information through a variety of terminal devices.

Second, social networking services create situations that spark multiple cooperative relationships. Study shows that facilitators of cooperation are diversity of parties (Kocak and Can, 2014); common values, experiences, commitment (Sá and Lee, 2012; Soetanto and Jack, 2013); trust (Bøllingtoft and Ulhøi, 2005; Bøllingtoft, 2012; Breznitz et al., 2018); and appropriate physical layout (Bøllingtoft and Ulhøi, 2005; Breznitz et al., 2018). Social networking services are powerful tools to promote multiple involvement of co-production and they facilitate network expansion. In another word, social networking services are an initiative situation to spark diversity of relationships. At the same time, social networking services could develop various virtual communities, by means of which formal and informal cooperation could occur (Kuhn et al., 2016). The formal and informal cooperation provide an additional channel for emotional exchange, which could cultivate common values and experiences and create friendship and common commitment. Furthermore, the function of word-of-mouth embedded in social networking services discourages parties from taking opportunist behaviors. Social networking services have become a mechanism to nurture trust among parties. Social networking services help to establish a non-hierarchical community, which could develop the pattern of bottom-up cooperation (Bøllingtoft, 2012). The bottom-up cooperation facilitate to smooth away physical constraints, which hinder the connections.

Third, social networks create situations that help financial services, entrepreneurial mentoring, and technical support to go smooth.

In the opinion of real options (Hackett and Dilts, 2004; Mian et al., 2016), financial service is the process of seeking profit maximizing in a dynamic environment. In order to change with the dynamic environment, tenants sought to develop hybrid networks (Van Rijnsoever et al., 2017). Hybrid networks are characterized of inherent resilience, delayable decision-making, expansive, and plausible solutions (Zardini et al., 2018). In one word, networks represent collaborations that share potential options, which increase total surplus of co-production. As hybrid networks help to develop situations that facilitate the creation, recognition, and execution of options and social networking services are a fundamental tool to establish and expand hybrid social networks, parties of financial services should resort to embed in social networking services extensively.

Entrepreneurial mentoring service either provide direct management consulting services such as mentoring on operation management, human resources management, and financial management or just play a broker to help tenants to gain access to external resource and knowledge. As social networking services could connect different boundaries of entrepreneurship ecology and create sparse, diversified social networks (Neumeyer et al., 2018), they can, thereby, facilitate entrepreneurship mentors to play the broker. Thus, social networking services could develop situations that facilitate entrepreneurial mentoring.

Technical support is essentially knowledge transfer (Cantù, 2017). Social networking services develop appropriate situations not only for exploitative learning, but also for collective learning (Hughes et al., 2007a). The exploitative learning helps tenants to master tacit knowledge and improves knowledge absorption capacity. At the same time, tenants and technical service providers could involve in explorative learning (Soetanto and Jack, 2016). By means of the function of information browsing of social networking services, it could facilitate them to develop a common knowledge background. Furthermore, the word-of-mouth function of social networking services positively strengthens the commitment of knowledge transfer, which could result to high willingness of involvement. In general, knowledge transfer depends on knowledge absorption capacity, common knowledge background, and willingness of knowledge transfer (Patton, 2014; Rubin et al., 2015). Then, social networking services could create appropriate situations that facilitate technical support.

According to contingency theory, to drive the growth of tenants, parties of co-production should adjust their behaviors with dynamic environment (Phan et al., 2005). Since social networking services create situations that facilitate the information dissemination, sparking connections, and realization of customized services, all the customized services should resort to being embed in social networking services.

Thus, we could speculate:

Hypothesis 1: Customized services result to behavior orientation of social networking services positively.

According to co-production theory by Rice (2002) and Eriksson et al. (2014), incubation management plays double roles in the process of co-production: (1) Directly participating in co-production as a service provider, according to willingness of participation and frequency of interaction—there are three patterns of involvement, namely, passive indirect participation, active indirect participation, and active continuous participation and (2) Just acting as broker to help tenants to connect with external networks. The function of real-time communications of social networking services facilitates incubator management to involve in active continuous participation in the process of co-production, which is the highest output pattern. In this circumstance, tenants could embed in multiple networks extensively (Pellinen, 2014; Blok et al., 2017). Then, they could resort to external supports to analyze their advantages and disadvantages of themselves, accurately understand market opportunities and threats, and avoid technological and market risks (Lamine et al., 2018).

On the other hand, social networking services facilitate incubator management to act as a broker. By means of multiple external networks developed by social networking services, tenants could spread information on their credibility (Morrish et al., 2019). Social networking services could magnify the reputation, so that tenants could attract more external service providers to involve in co-production passively and then tenants could develop competitive networks of cooperation (Pettersen et al., 2016; Eveleens et al., 2017). The multiple effects of passive parties and competitive networks facilitate incubator management to play a competent broker, which promote the growth of incumbent firms in the end.

At the same time, social networking services also weaken the constraints of social norms and enterprise culture on entrepreneurship. Tenants located on incubator typically pursue knowledge commercialization by means of new technologies, new business models, and new products (M’Chirgui et al., 2018). This brings endogenous conflicts with the existing social norms and cultural traditions. It, in turn, may lead to mutual distrust, conflicts in communication, and incompatible behavior pattern, which could hinder the process of co-production. While the virtual community developed by social networking services is a completely new situation for collaboration (Ariza-Montes and Muniz, 2013), by means of which tenants could eliminate the negative effects of existing social norms and enterprise culture in some extent. In the opinion of external environment, social networking services could develop an appropriate situation to facilitate the growth of client firms.

Next, we focus on community-level social capital, which comprises bonding social capital, bridging social capital (Redondo and Camarero, 2019), as well as linking social capital.

Bonding social capital refers to close relationships between network nodes. These relationships originate from mutual trust and commitment. In turn, mutual trust and commitment intensify the close relationships between them. In additional, word-of-mouth effect of social networking services removes the opportunists from the cooperative networks, so the balance of game is to further keep mutual commitments. As mutual commitments intensify the close relationships, social networking services could strength bonding social capital (Branstad and Saetre, 2016).

Bridging social capital refers to the connections with external community. It is an important channel for information flow between heterogeneous nodes. As social networking services have the function by means of which tenants could connect with external nodes passively, tenants could develop more connections with external community (Apa et al., 2017) and then client firms could enrich bridging social capital in the existing entrepreneurship ecology.

Linking social capital refers to those connections between distinct hierarchies in a community. The transhierarchical relationships allow tenants to connect with government agencies, incubator management, client firms, and other entrepreneurs, which facilitate tenants to obtain useful information and resources outside the formal hierarchies. Since social networking services could develop a kind of virtual community, which is a non-hierarchy organization, social networking services should be an effective measure to develop transhierarchical connections. The relationships embedded in transhierarchical connections help to smooth away organization gap, orientation of bureaucracy, as well as differentiation of identification (Carayannis and Maximilian, 2005). Thus, social networking services help tenants to gain more linking social capital, which could facilitate the growth of tenants.

We could speculate:

Hypothesis 2: The behavior orientation of social networking services could promote the growth of tenants.

Customized service is a kind of demand-driven processes including three subprocesses—information dissemination of demand, sparking of specific relationships, and realization of services. Customized service focuses on demands of customer-oriented, which is other from supply oriented logic (Tello et al., 2012) and its value lies on meeting specified commands. As specified demands pull the process of customized services, tenants have endogenous initiatives to take active part in co-production. The passive involvement of co-production should improve the quality of services and facilitate the growth of tenants.

Next, as the heterogeneity of needs increases, customized services become more effective. Owing to distinct technologies, entrepreneurs, and external environments, demands of tenants are highly heterogeneous. Even we divide customized services into three subcategories: financial services, entrepreneurial mentoring, and technical assistance, the heterogeneous of demand is still obvious. Study shows that services, which could meet the idiosyncratic needs of consumers, lead to higher customer satisfaction (Vanderstraeten et al., 2016). Then, customized services promote the growth of tenants by means of higher customer satisfaction (Cooper et al., 2012).

Third, owing to distinct resource endowments, technology, and life cycle (Fukugawa, 2017), tenants resort to dynamic situations to deal with changeable environment. Customized services can help tenants to develop appropriate situations, which could change with external environment (Elfring and Hulsink, 2007). At the same time, customized services bring about diversified networks to help tenants to make use of opportunities and deal with threats (Huggins and Thompson, 2015). Furthermore, customized services have no fixed “roadmaps” and it will facilitate tenants to change their organizations in a dynamic manner. So, customized services facilitate tenants to change with environment timely and it could promote the growth of tenants (Junaid Ahmad, 2014).

Thus, we could speculate:

Hypothesis 3: Customized services positively promote the growth of tenants.

Customized services can directly promote the growth of tenants. In another way, customized services lead to the behavior orientation of social networking services; at the same time, behavior orientation of social networking services facilitates the growth of tenants.

Then, we could speculate:

Hypothesis 4: Social networking services mediate the relationship between customized services and the growth of tenants.

**FIGURE 2:**
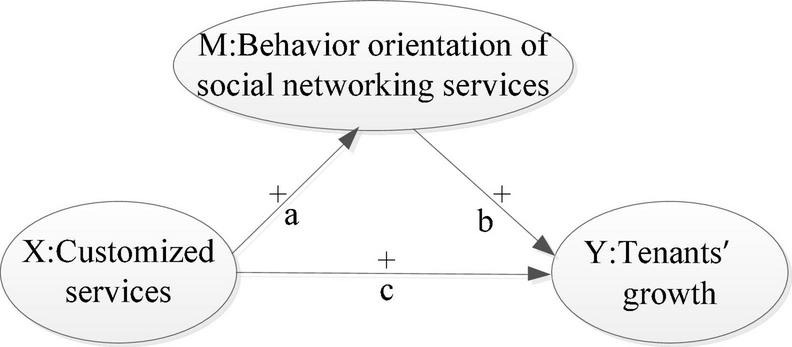
Research model. (a–c) Represent three paths which would be explored, while “+” represents the expected positive impact.

The research model is shown in Figure 2.

## Methodology

### Sample and Data Collection

Incubator is a part of national innovation systems and it is subjected to regional economic development. In order to test of research model, it is necessary to smooth away sample biases of regions. So, this study investigated state-level business incubators located in the five regions according to the general administrative division of China: Northeast, North, East, Central, South, and West. A total of 200 incubators were selected from the list of incubators on the website of the Torch High-Tech Industrial Development Center of the Ministry of Science and Technology^2^; accordingly, 40 incubators are selected from each region randomly. The samples are selected from state-level incubator because the operations of those incubators are in good manner; data compiled are complete and statistical methods of data are plausible.

Additional, Torch High-Tech Industrial Development Center agreed to backup the surveys as we have cooperated with the Strategy Research Institute of Wuhan East Lake High-Tech Development Zone for a long time, while the Wuhan East Lake High-Tech Development Zone and the Torch High-Tech Industrial Development Center have official collaboration. So, we were granted timely access to the annual statistical reports from official agencies responding to investigation about China incubator. Those information and data could be an alternative to those from surveys.

**FIGURE 3:**
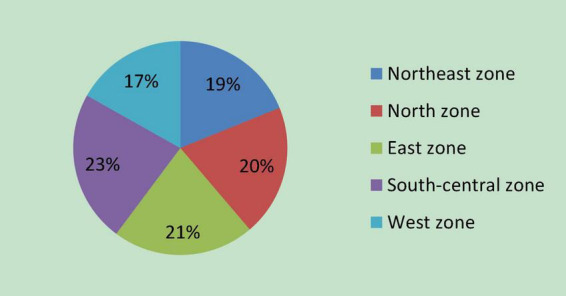
Regional distribution of the samples.

In the name of the Torch High-Tech Industrial Development Center, we distributed 200 surveys through e-mail, WeChat, and an online chat APP (QQ). The survey states that this study aimed at assistance of policymaking explicitly and it was endorsed by the Strategy Research Institute of the Wuhan East Lake High-Tech Development Zone, so we collected 142 complete responses, 37 incomplete responses, and 21 surveys have no responses at last. We also distributed surveys face-to-face by means of annual meeting and special conference and collected 54 responses. Thus, the total valid samples are 196. Figure 3 shows the regional distribution of 196 samples. The samples from South-Central zone and East zone are slightly more. In general, the samples are evenly distributed in five zones and have no regional bias.

### Measurement of Variables and Reliability and Validity Analysis

Mathematical and psychological theories and empirical analyses have witnessed that 7-point Likert scale is plausible to measure the extent of human behavior and psychological inclination (Podsakoff et al., 2003). The measurement items have been widely used in those articles, which have been published on prestige international journals.

To measure customized services, which others perform for tenants, we adopted the 7-point Likert scale item, which has been employed by Skaggs and Huffman (2003) and Skaggs and Youndt (2004) as well as by Vanderstraeten et al. (2016).

On the other way, the articles “Networked Incubators” (Hansen et al., 2000) and “Stimulating Dynamic Value: Social Capital and Business Incubation as a Pathway to Competitive Success” (Hughes et al., 2007b) have cracked the measurement of social networking behavior. It has been measured by 7-point Likert scale as well and we adopted it according to the context of incubator.

Additionally, growth of tenants has been still estimated by 7-point Likert scale in an article published on “Journal of Engineering and Technology Management” (Vanderstraeten et al., 2016). Other than the mature ventures, it is well accepted and widely employed to measure the growth of a newborn in terms of subjective evaluation. We adopted those items of 7-point Likert scale according to the growth of tenants located on incubator.

Table 1 shows the origin of items in detail. The items were adapted in order to facilitate the responders whose native language is Chinese, but the changed items remain the original meaning. We employed a 7-point Likert scale to measure these items ranging from “1 = I strongly disagree” to “7 = I strongly agree,” while from 1 to 7 account for corresponding extent of agreement.

**TABLE 1:** Items and reliability analysis.

**Variables**	**Items**	**Items adapted from**	**Corrected item-total correlation**	**Cronbach**’**s alpha if item deleted**	**Cronbach**’**s alpha**	**Composite reliability (CR)**
Customized service (ξ _1_)	*x*_*11*_ The service provider obtains a large amount of information about the tenants prior to services through investigation and consultancy	Skaggs and Huffman, 2003; Skaggs and Youndt, 2004; Vanderstraeten et al.,2016	0.880	0.955	0.962	0.966
	*x*_*12*_Distinct services are provided according to demands of different stages of growth		0.895	0.952		
	*x*_*13*_ Portfolios of services are provided based on the specified demands of tenants in a certain stages of growth		0.897	0.952		
	*x*_*14*_ In the whole process, the service providers keep close contact with tenants to make sure tenants’ demands		0.893	0.952		
	*x*_*15*_The service provider is capable of providing real-time and dynamic services based on the specified demands		0.895	0.952		
Behavior orientation of social networking services (η_1_)	*y*_*11*_Parties involved in services always want to be a member of networks by means of social networking services	Hansen et al., 2000; Hughes et al.,2007b	0.883	0.947	0.957	0.965
	*y*_*12*_ Parties involved in services confirm that it is necessary to involve in networks by means of social networking services		0.901	0.941		
	$y{}_{13}$ Parties involved in services often attempt to obtain all kinds of valued assistance by means of social networking services		0.894	0.943		
	$y{}_{14}$ Parties involved in services often gain access to diversified formal and informal collaborations by means of social networking services		0.903	0.940		
Tenants’ growth (η_2_)	$y{}_{21}$ How many tenants which admitted to locate on incubator in 2013 have graduated by December 31, 2016? The proportion (%) is ____	Vanderstraeten et al.,2016	0.881	0.925	0.944	0.952
	$y{}_{22}$ As to tenants which admitted to locate on incubator in 2013 have graduated by December 31, 2016, how many tenants have achieved an increase in employees? The proportion (%) is____		0.878	0.924		
	$y{}_{23}$ As to tenants which admitted to locate on incubator in 2013 have graduated by December 31, 2016, how many tenants have achieved an increase in sales revenue? The proportion (%) is ____		0.877	0.924		
	$y{}_{24}$ As to tenants which admitted to locate on incubator in 2013 have graduated by December 31, 2016, how many tenants have achieved an increase in profit? The proportion (%) is ____		0.840	0.937		

The reliability of the variables was analyzed using reliability and composite reliability analysis (Table 1). The values of the Cronbach’s alpha and composite reliability are larger than the threshold of 0.7 and the corrected item-total correlation (CITC) is greater than 0.5 at the same time of the Cronbach’s alpha, if item deleted decreases. This confirms the reliability of items.

Validity analysis includes: (1) Face validity: this refers to whether the words that described the items are accurate and appropriate. Science those items were adapted from existing literatures, they are of good face validity, (2) Convergent validity: this refers to whether items are highly correlated when they measure the same variable. The convergent validity of the items can be evaluated by analyzing their standardized loading and average variance extracted (AVE) of items. The items exhibit good convergent validity, if their standardized factor loading is greater than 0.70 and the AVE of variables is greater than 0.50. Table 2 shows that all the items exhibit good convergent validity; (3) Discriminatory validity: this refers to whether the correlation between the variables is low. When the variables AVE in the model is greater than the square of their correlation coefficient (or the square root of the AVE is greater than the correlation coefficient), each item only loads on the variable and has no cross-loading; thus, the variables exhibit good discriminatory validity. Comparing the correlation coefficients and AVEs of the latent variables in the model (Table 3) and conducting a factor analysis of the items (Table 4), it reveals that all the items exhibit good convergent validity. Moreover, all the items load on the three independent factors and the eigenvalue of three factors is greater than 1.0; they account for 87.352% of the total variation. This confirms that the sample has no common method biases (Podsakoff et al., 2003).

**TABLE 2:** Confirmatory factor analysis.

**Variables**	**Items**	**Standardized loading**	**AVE**	**Goodness of fit statistics**
Customized service (ξ _1_)	$x{}_{11}$	0.920***	0.853	
	$x{}_{12}$	0.930***		
	$x{}_{13}$	0.920***		χ^2^ = 142.76
	$x{}_{14}$	0.930***		df = 62
	$x{}_{15}$	0.920***		χ^2^/df = 2.303
Behavior orientation of social networking services (η_1_)	$y{}_{11}$	0.930***	0.874	RMSEA = 0.082 GFI = 0.900 NFI = 0.970
	$y{}_{12}$	0.940***		IFI = 0.980
	$y{}_{13}$	0.930***		CFI = 0.980
	$y{}_{14}$	0.940***		RFI = 0.970
Tenants’ growth (η_2_)	_21_	0.920***	0.833	
	$y{}_{22}$	0.940***		
	$y{}_{23}$	0.930***		
	$y{}_{24}$	0.860***		

***p < 0.001.

**TABLE 3:** Correlation coefficient and average variance extracted (AVE) of variables.

**Variables**	**AVE**	ξ _1_	η _1_	η _2_
ξ _1_	0.853	**0.924**		
η _1_	0.874	0.760	**0.934**	
η _2_	0.833	0.550	0.500	**0.912**

*The bold numbers above the diagonals are value of square roots of AVE; the other numbers are the correlation coefficients between the variables.*

**TABLE 4:** Factor loading of items and cumulative variance.

**Items**	**Factor**		
	**1**	**2**	**3**
x11	**0.806**	0.359	0.271
x12	**0.860**	0.302	0.222
x13	**0.836**	0.362	0.210
x14	**0.853**	0.293	0.252
x15	**0.795**	0.459	0.192
y11	0.364	**0.830**	0.224
y12	0.326	**0.868**	0.193
y13	0.368	**0.845**	0.191
y14	0.378	**0.849**	0.177
y21	0.233	0.139	**0.896**
y22	0.215	0.167	**0.892**
y23	0.191	0.197	**0.891**
y24	0.177	0.178	**0.876**
Cumulative variance (%)	**62.809**	**79.279**	**87.352**

*Extraction method: Principal component analysis.*

*Rotation method: Varimax with Kaiser normalization.*

*Rotation converged in 7 iterations.*

*The bold values respond to the factor loading of the left items.*

## Data Analysis and Hypothesis Testing

### Model Testing

All the items in the theoretical model have a minimum loading of 0.869 and are significant. This confirms that the model meets the baseline of goodness of fit statistics. Furthermore, the reliability of the items is greater than 0.5, the composite reliability of the items is greater than 0.7, and the AVE of the variables is greater than 0.5. This confirms that the theoretical model has an appropriate structural fitness (Tables 1, 2). Table 5 shows the goodness of fit statistics. According to a rule of thumb, all the parameters are acceptable besides root mean square error approximation (RMSEA), but it is just a little high. So, the mediation model could be used to test the hypotheses.

**TABLE 5:** Goodness of fit statistics.

**Goodness of fit statistics**	**df**	**x^2^/_df_**	**RMSEA**	**GFI**	**NNFI**	**CFI**	
Mediation model	387.63	62	625	0.164	0.82	0.93	0.94

### Analysis of Mediating Effect

**FIGURE 4:**
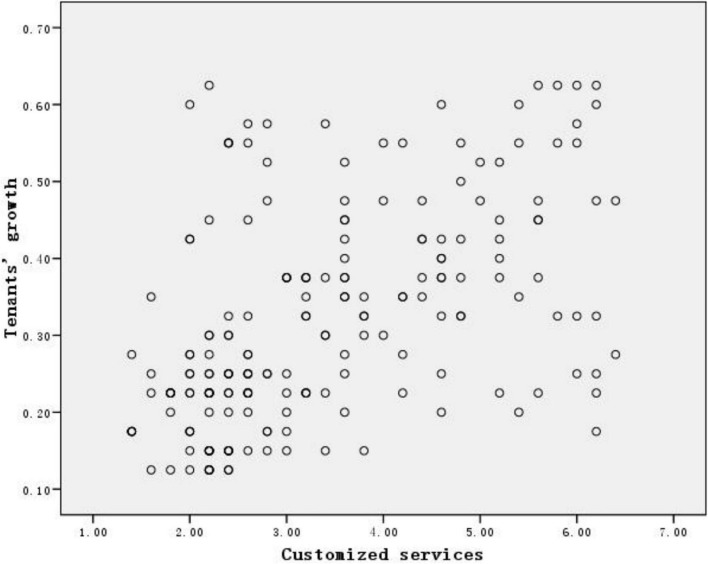
Scatter diagram.

First, we should ascertain whether there are positive relationship between customized services and growth of tenants. The scatter diagram of the two variables shows that they are positively correlated (Figure 4). So, we could employ regression analysis in detail.

Table 6 shows that the coefficient between the two variables is significant at the 0.001 level.

**TABLE 6:** Regression coefficients.

**Model**		**Unstandardized coefficients**		**Standardized coefficients**	***t***	**Sig.**
		**B**	**Std. Error**	**Beta**		
1	(Constant)	0.149	0.023		6.481	0.000
	Tenants’ growth	0.050	0.006	0.501	8.061	0.000

*^*a*^Dependent variable: customized services.*

If coefficients of path a and b (Figure 2) are both significant, then the relationship between x and y is a partial mediation. In this case, it is necessary to test the coefficient of path c in the next place. If coefficient of path c is not significant, then the variable M totally mediates the relationship between X and Y and it is a full mediation process (Judd and Kenny, 1981). If coefficient of path c is significant, then the variable M partially mediates the relationship between X and Y and it is a partial mediation process (Shah and Goldstein, 2006). If either coefficient path a or path b is significant, then they must to be subjected to the Sobel test. If the coefficient of the Sobel test is significant, then the mediating effect of M is significant (Sobel, 1982).

**FIGURE 5:**
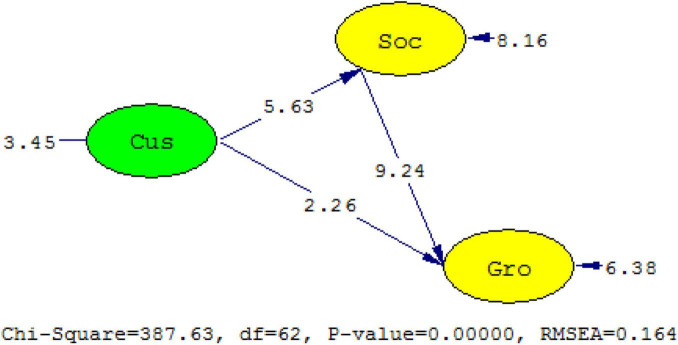
*T*-values of the model. Cus-ξ _1_, customized service; Soc-η_1_, behavior orientation of social networking services; Gro-η_2_, growth of tenants.

According to the table of the *t*-test, the *t*-value is 3.460 when degree of freedom is 60 and significance level is 0.001. The degree of freedom of the mediation model is 62, while the *t*-values of coefficient of path a and path b are 5.63 and 9.24, respectively (Figure 5), which are notably greater than 3.460. Thus, they are significant at the 0.001 level. While the *t*-value of path c is 2.26, it is not significant at the 0.001 level. This confirms that behavior orientation of social networking services has a full mediating effect between customized services and growth of tenants.

## Research Conclusion and Management Implications

### Conclusion

**FIGURE 6:**
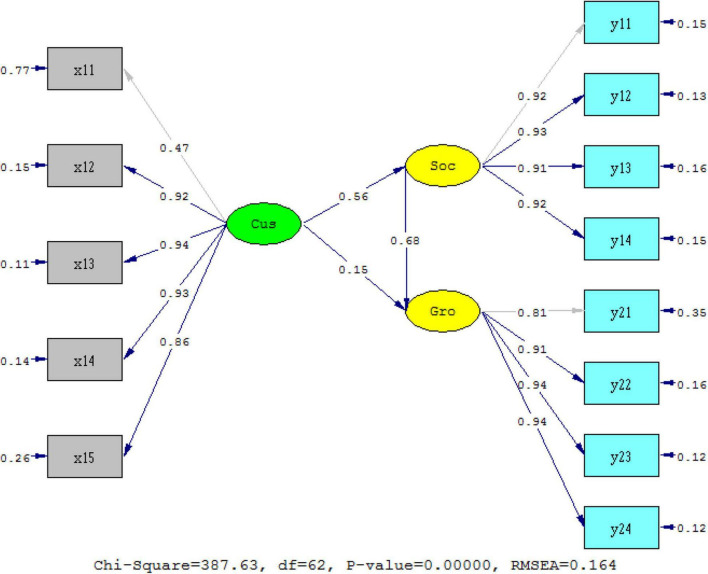
Standardized solution of path coefficients. Cus-ξ _1_, customized service; Soc-η_1_, behavior orientation of social networking services; Gro-η_2_, growth of tenants.

This study focuses on the relationship between customized services, behavior orientation of social networking services, and growth of tenants. Our conclusions can be summarized as follows: customized services directly facilitate the growth of tenants (β = 0.501, *p* = 0.001). While we focus on the relationship among customized services, behavior orientation of social networking services, and growth of tenants, we found that customized services facilitate the growth of tenants via behavior of social networking services; at the same time, customized services had no direct impact on the growth of tenants. We could draw the conclusion that behavior of social networking services could fully mediate the relationship between customized services and growth of tenants. As shown in Figure 6, coefficients of path a and path b are 0.56 and 0.68, respectively, and they are both significant at 0.001 level, while coefficient path c is not significant, even at 0.05 level. Thus, the mediating effect is *M* = 0.56 × 0.68.

It could be concluded that though customized incubation services directly impact the growth of tenants, the behavior orientation of social networking services fully mediates the effect of customized incubation service (Wu and Si, 2018). As heterogeneous network accesses have become one of the primary incubator services (Bartosik-Purgat and Ratajczak-Mrożek, 2018) and tenants are inclined to fully embed in networking, venture founders would have to resort to social networking services to fulfill their commitments because of its convenience and efficiency (Cohen, 2006).

As a result, once social networking is employed to facilitate the customized services, involvement of social networking of the founder would fully determine the effect of customized services (Li and Tang, 2019). Unwillingness to interaction by means of social networking would inevitably lock in the poor growth. On the contrary, tenants that have engaged in social networking to learn, communicate, search information, as well as gain all the sorts of incubation services are liable to make full use of customized incubation services (Albahari et al., 2018). Though the fundamental force to drive the growth of tenants is customized incubation services, the more direct facilitator is orientation of social networking behavior (Beyhan and Findik, 2018). In theory, we speculate that social network behavior determines not only how the services could be perform for tenants, but also what virtual contents of the customized services could be delivered. Social networking might totally reform the modality of co-production as well as the general connotation of incubation services (Bouncken and Reuschl, 2018).

### Management Implications

(1) Incubation services must be deeply embedded in social networking services.

The mediating effect of behavior of social networking services indicates that social networks are most important measures to facilitate customized services. It is necessary to develop and improve infrastructure of mobile network, which could set the baseline of social networking services. We should promote all the co-production to embed in social networking services extensively (Theodoraki et al., 2018). First, we should improve the infrastructure of mobile network. Nowadays, China has realized mobile network full coverage in state-level high-tech development districts. Government agency and incubation management have realized the impact of social networking services. So, it is the priority that we should take effective measures to push the process of 5G commercialization, thus we could enter a new era of “customized services in a real-time manner” by means of the function of zero time-delay of 5G mobile network. Next, incubator management should develop diversified scenes, which could stimulate the whole process to embed in social networking services extensively, for example, it should be helpful to arrange the whole workflow to be finished by means of social networking services. Finally, it is necessary to develop specified social network software to attract all the parties of co-production to involve in social networking services actively. This software should be different from universal social media software; it should be developed based on the specified scenes of customized services, so as to promote all the parties to interact by means of social networking services (Kumar et al., 2017).

(2) Incubator management should improve the capability to deal with big data embedded in social networking services.

Social networking services are accompanied by emerging big data. If incubator management could make use of big data to guide the customized services, it could gain competitive advantage (Eggers and Kaul, 2018). So, it is necessary to setup a new department to deal with big data specially, even incubator management could purchase professional services to deal with big data such as collecting big data, storage of big data, analysis of big data, and visualization of big data. By doing this, incubator management could integrate big data management into the basic services of incubator. It is also important to advocate data sharing of incubators, even pay for it (Díez-Vial and Montoro-Sánchez, 2017). Government agency responsible for incubator management could develop an open database, which would become a trustworthy source of data and incubator management could drive customized services by means of the database.

(3) Entrepreneurial ecosystems should be intensively embedded in social networks.

Entrepreneurial ecosystems represent a diverse set of interdependent actors within a geographic region that influence the formation and eventual trajectory of the entire group of actors and potentially the economy as a whole. Entrepreneurial ecosystems evolve through a set of interdependent components, which interact to generate new venture creation over time (Pugh et al., 2019). As entrepreneurial ecosystem is a kind of macroenvironment in which tenants grow, the social networking services embedded in entrepreneurial ecosystem underpin how the tenants could make use of social networking. If we could promote agents of entrepreneurial ecosystem, such as governments, universities, tenants, service providers, agency, etc., to be linked by means of special social networking software, it could cultivate appropriate external macroenvironments for tenants to make use of social networking services (Seno Wulung et al., 2017).

## Data Availability Statement

The raw data supporting the conclusions of this article will be made available by the authors, without undue reservation.

## Ethics Statement

The studies involving human participants were reviewed and approved by the Ethics Committee of Hubei University. The patients/participants provided their written informed consent to participate in this study. Written informed consent was obtained from the individual(s) for the publication of any potentially identifiable images or data included in this article.

## Author Contributions

LZ developed the research model, conducted the analysis, co-drafted the manuscript, collected the data, and edited the manuscript. YZ co-drafted the manuscript and responded to the reviews. Both authors contributed to the article and approved the submitted version.

## Conflict of Interest

The authors declare that the research was conducted in the absence of any commercial or financial relationships that could be construed as a potential conflict of interest.

## Publisher’s Note

All claims expressed in this article are solely those of the authors and do not necessarily represent those of their affiliated organizations, or those of the publisher, the editors and the reviewers. Any product that may be evaluated in this article, or claim that may be made by its manufacturer, is not guaranteed or endorsed by the publisher.

## Footnotes

1
https://en.wikipedia.org/wiki/Social_networking_service

2
https://fuwu.most.gov.cn/
